# JPred4: a protein secondary structure prediction server

**DOI:** 10.1093/nar/gkv332

**Published:** 2015-04-16

**Authors:** Alexey Drozdetskiy, Christian Cole, James Procter, Geoffrey J. Barton

**Affiliations:** Division of Computational Biology, College of Life Sciences, University of Dundee, Dundee, DD1 5EH, UK

## Abstract

JPred4 (http://www.compbio.dundee.ac.uk/jpred4) is the latest version of the popular JPred protein secondary structure prediction server which provides predictions by the JNet algorithm, one of the most accurate methods for secondary structure prediction. In addition to protein secondary structure, JPred also makes predictions of solvent accessibility and coiled-coil regions. The JPred service runs up to 94 000 jobs per month and has carried out over 1.5 million predictions in total for users in 179 countries. The JPred4 web server has been re-implemented in the Bootstrap framework and JavaScript to improve its design, usability and accessibility from mobile devices. JPred4 features higher accuracy, with a blind three-state (α-helix, β-strand and coil) secondary structure prediction accuracy of 82.0% while solvent accessibility prediction accuracy has been raised to 90% for residues <5% accessible. Reporting of results is enhanced both on the website and through the optional email summaries and batch submission results. Predictions are now presented in SVG format with options to view full multiple sequence alignments with and without gaps and insertions. Finally, the help-pages have been updated and tool-tips added as well as step-by-step tutorials.

## INTRODUCTION

Knowledge of a protein's three-dimensional structure is central to understanding the protein's detailed function. Although recent developments in structural biology (1–4) have led to an acceleration in the rate of three-dimensional structure determination by X-ray crystallography, nuclear magnetic resonance and 3D-EM techniques, in January 2015 there were still just 105 732 protein structures known (http://www.ebi.ac.uk/pdbe) (5) compared to almost 90 million sequences (http://www.ebi.ac.uk/uniprot/TrEMBLstats) (6). The routine use of massively parallel DNA sequencing technologies today means knowledge of protein sequences will continue to outpace structural biology for the foreseeable future. As a consequence, there is a need for accurate methods to predict structural and functional features from the amino acid sequence. Over the last 30 years, techniques to predict the three-state secondary structure of the protein (α-helix, β-strand and coil: i.e. all other states) have increased in accuracy from around 50% in 1983 (7) to over 80% today (8–11) which is close to the estimated maximum for prediction from multiple alignment (12). Although knowledge of the secondary structure alone is not as useful as a full three-dimensional model, secondary structure predictions provide important constraints for fold-recognition techniques (13–17) as well as in homology modelling (18,19), *ab initio* (20–24) and constraint-based tertiary structure prediction methods (25–27). Secondary structure predictions can also help in the identification of functional domains and may be used to guide the rational design of site-specific or deletion mutation experiments.

Although hundreds of papers have been published describing methods for protein secondary structure prediction, three of the most widely used are JPred, PSIPRED and PredictProtein. JPred (v. 3.0) (11) gave 81.5% three-state accuracy (Q_3_), PSIPRED v.3.0 (28) reported accuracy of 81.4%, while the current PSIPRED V 3.2 server, which includes a broad suite of prediction algorithms, quotes 81.6%. (http://bioinf.cs.ucl.ac.uk/psipred). There is no recent blind prediction test for the PROFphd secondary structure prediction algorithm in the PredictProtein (29) secondary structure prediction method, though the earlier PROFsec reported 76% (30).

In this paper we summarize the current performance and features of the upgraded JPred server (JPred4) which incorporates the secondary structure and solvent accessibility prediction program JNet v.2.3.1.

## MATERIALS AND METHODS

The basic usage pattern for JPred4 is the same as for JPred3 (11). The user can submit a single protein sequence, a multiple sequence alignment (MSA) or a batch of single protein sequences for prediction. Results are returned either interactively through a web page or as a summary email that directs the user to results on the JPred4 website.

**Figure 1.**
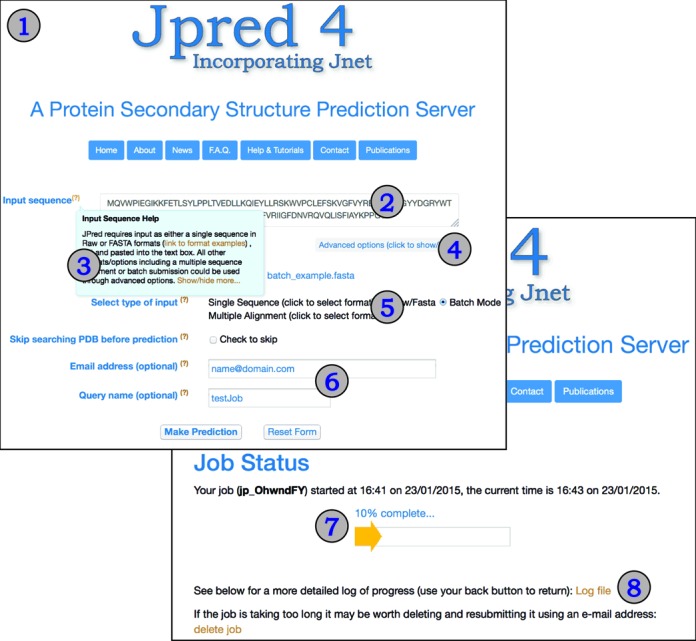
(1) Screenshot of the JPred4 job submission page with single sequence submission field (2) and an example of a tool-tip message (3). Advanced options are opened on request (4) and include input file upload, format selection (5) as well as optional email and query name fields (6). (7) Job progress page with access to the detailed job run log file (8).

The look and feel of the JPred4 web server has been changed significantly compared to JPred3 by embracing contemporary web technologies, the Bootstrap framework (www.getbootstrap.com/) and custom JavaScript. These changes allow smoother user interaction through the use of ‘tooltips’ that pop up to present help on each option in an easy-to-read form without the need to leave the page. The Bootstrap framework provides a modern look and feel to the website as well as improving usability on devices such as tablets and phones with different screen sizes and resolutions. Figure 1 illustrates the appearance of the advanced submission page showing the use of tooltips to get help about each option. As well as updates to the help pages, step-by-step tutorials with screenshots are a new addition that helps users to obtain maximum benefit from the JPred4 server.

### Prediction algorithm

As with JPred3, JPred4 makes secondary structure and residue solvent accessibility predictions by the JNet algorithm (11,31). However, in JPred4, the JNet 2.0 neural network-based predictor has been retrained to make JNet 2.3.1 by 7-fold cross-validation using one representative for each of the 1358 SCOPe/ASTRAL v.2.04 superfamily domain sequences (32). Multiple alignments for each sequence were built by PSI-BLAST (33) through searching UniRef90 v.2014_07 (34). In addition to retraining, the HMM building step in JNet was updated to HMMer 3 (35) and some improvements were made to the code to simplify management and future algorithmic developments. The final accuracy of JNet 2.3.1 was assessed in a blind test on 150 sequences from 150 superfamilies not used in training. The 150 superfamily sequences were selected to reproduce a similar distribution of secondary structure compositions as the training structures in order to avoid biasing the reported accuracy of the blind test results. On the blind test, the average secondary structure prediction Q_3_ score increased to 82.0% from 81.5% for JNet v.2.0, and solvent accessibility prediction accuracy rose to 90.0, 83.6 and 78.1% from 88.9, 82.4 and 77.8% for JNet v.2.0 for each of >0, >5 and >25% relative solvent accessibility thresholds.

### JPred4 results reporting

JPred3 has been widely used in teaching and integrated into many bioinformatics pipelines across the world. Accordingly, in order to maintain support for legacy courses and scripts, the results options in JPred4 include all the original formats and styles (PDF, HTML, etc.) as well as the intermediary processing files. In addition to these outputs, JPred4 reports have been enhanced to include more visualization options and to present a complete picture of the alignment generated for prediction including all insertions.

**Figure 2.**
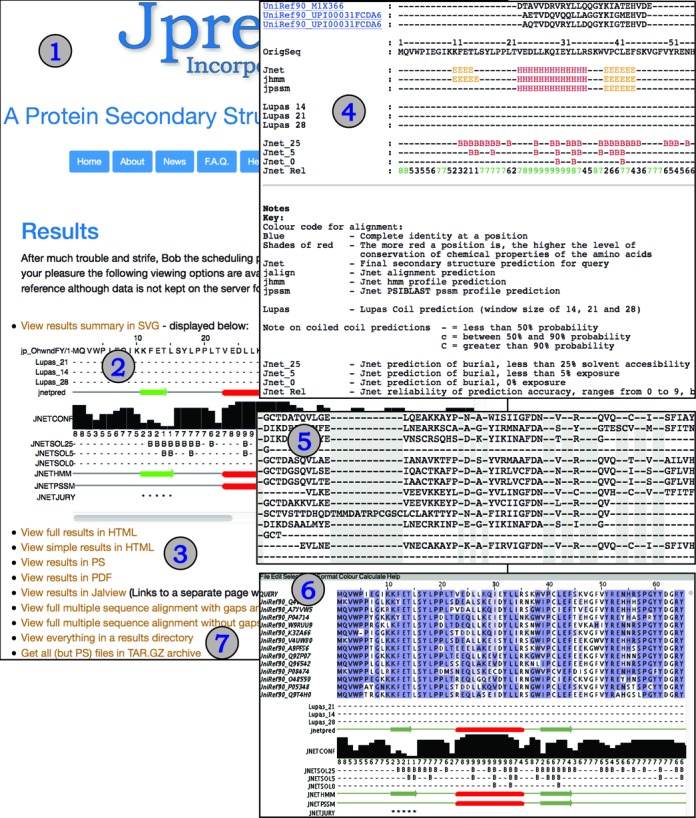
JPred4 results summary page (1) with the results of predictions presented in SVG (2). Links to detailed and simple reports in coloured HTML/PS/PDF formats (3). Example summary in HTML format is shown in (4) as well as the new addition of full multiple sequence alignments with and without gaps/insertions (5). On a separate linked page the user is able to run the Jalview applet (6) which allows a more sophisticated and interactive method of viewing the prediction results. Links to all the details for the prediction and an archive of the results are also available (7).

**Figure 3.**
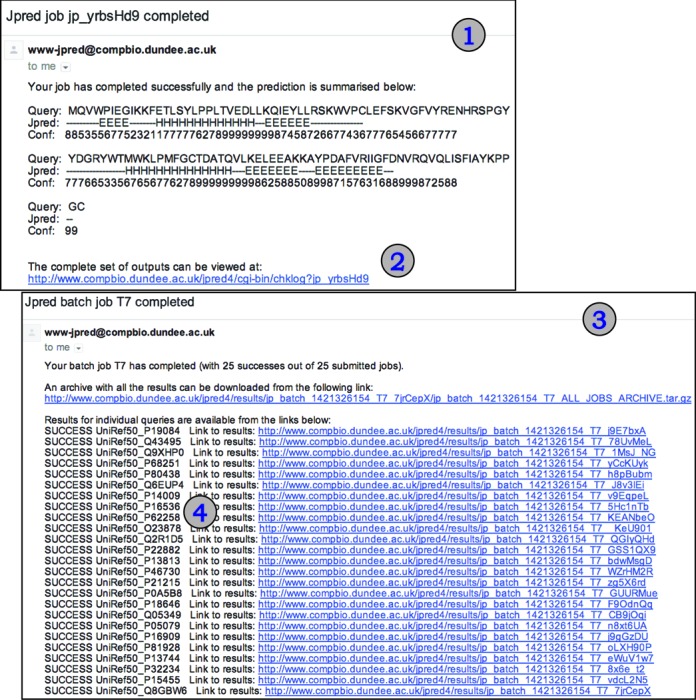
(1) Illustration of a single sequence job submission secondary structure prediction results summary email with link to full result details (2). (3) Illustration of a batch submission email summary with overall and per job (4) details that give links to individual predictions and an archive with all results for all sequences submitted in the batch.

Figure 2 summarizes the main results page while Figure 3 shows examples of summary emails returned to a user for single or batch sequence submissions. Unlike previous versions of JPred, the primary visualization of a JPred4 prediction result is a scrollable SVG image. The SVG is generated by Jalview 2.9 (www.jalview.org) (36) run in command-line mode as part of the JPred4 web server processing pipeline so users do not need to run Jalview on their own computers. However, the JalviewLite Java applet result page is still provided for users working with Java-enabled browsers who prefer direct access to Jalview's sophisticated functions.

In all previous versions of JPred, the alignment returned showed the full-length query sequence without gaps necessary to accommodate insertions in sequences returned from the PSI-BLAST search. JPred4 introduces options to view the full multiple alignment including all residues in all sequences or download it for further analysis. For users who have local installations of Jalview (36), Jalview feature files are provided to allow easy annotation and analysis of the alignment and predictions.

In JPred3, a batch job with multiple query sequences would return separate emails for each query. JPred4 condenses these messages into a single email with a summary of success/failure for each sequence (Figure 3) in the batch and a compressed archive of all the predictions.

All JPred4 jobs are currently stored on the server for 5 days.

### Time required to complete predictions

The median time for a JPred4 prediction to return results is 5 min calculated over a recent 50 000 consecutive predictions performed by end-users in the autumn of 2014. However, the server can accommodate jobs of up to 3-h duration. Most of the time is spent in the PSI-BLAST search phase which is avoided if the user submits a pre-existing MSA. MSA predictions typically return results within a few seconds.

In summary, the JPred server has been upgraded to provide a richer user experience and to include more accurate secondary structure and solvent accessibility predictions from the JNet 2.3.1 algorithm.
